# Reward Associations Reduce Behavioral Interference by Changing the Temporal Dynamics of Conflict Processing

**DOI:** 10.1371/journal.pone.0053894

**Published:** 2013-01-10

**Authors:** Ruth M. Krebs, Carsten N. Boehler, Lawrence G. Appelbaum, Marty G. Woldorff

**Affiliations:** 1 Center for Cognitive Neuroscience, Duke University, Durham, North Carolina, United States of America; 2 Department of Experimental Psychology, Ghent University, Ghent, Belgium; 3 Department of Psychiatry and Behavioral Sciences, Duke University, Durham, North Carolina, United States of America; 4 Department of Psychology and Neuroscience, Duke University, Durham, North Carolina, United States of America; University of California, Davis, United States of America

## Abstract

Associating stimuli with the prospect of reward typically facilitates responses to those stimuli due to an enhancement of attentional and cognitive-control processes. Such reward-induced facilitation might be especially helpful when cognitive-control mechanisms are challenged, as when one must overcome interference from irrelevant inputs. Here, we investigated the neural dynamics of reward effects in a color-naming Stroop task by employing event-related potentials (ERPs). We found that behavioral facilitation in potential-reward trials, as compared to no-reward trials, was paralleled by early ERP modulations likely indexing increased attention to the reward-predictive stimulus. Moreover, reward changed the temporal dynamics of conflict-related ERP components, which may be a consequence of an early access to the various stimulus features and their relationships. Finally, although word meanings referring to potential-reward colors were always task-irrelevant, they caused greater interference compared to words referring to no-reward colors, an effect that was accompanied by a relatively early fronto-central ERP modulation. This latter observation suggests that task-irrelevant reward information can undermine goal-directed behavior at an early processing stage, presumably reflecting priming of a goal-incompatible response. Yet, these detrimental effects of incongruent reward-related words were absent in potential-reward trials, apparently due to the prioritized processing of task-relevant reward information. Taken together, the present data demonstrate that reward associations can influence conflict processing by changing the temporal dynamics of stimulus processing and subsequent cognitive-control mechanisms.

## Introduction

Over the past decade, a number of human neuroimaging studies have demonstrated that associating rewards with specific stimuli can facilitate behavior, which has been shown to be paralleled by modulations in brain regions implicated in the processing of reward value [1], [2], as well as in regions implicated in attentional control [3]–[6]. These modulatory effects of reward are of particular interest in cognitively challenging conditions, such as when conflicting stimulus inputs trigger competing response tendencies. Yet, the current knowledge about the mechanisms by which reward associations influence neural processing in the context of such conflicting stimuli is limited.

In the absence of reward, participants are typically slower and commit more errors when faced with conflicting stimulus inputs as compared to non-conflicting ones. For example, in the color-naming Stroop task, responses are slower when the font color of a target word does not match its semantic meaning (e.g. “RED” written in green font) compared to when the font color matches the semantic meaning [e.g. “RED” in red font, [7]]. These behavioral costs are thought to arise from an automatic co-activation of the response to the task-irrelevant word meaning that needs to be overcome in order to correctly respond to the task-relevant font color [8]–[12]. The processing, and ultimately the resolution, of this interference is thought to rely critically on frontal brain regions involved in attentional and cognitive control [13]. The present study aims at elucidating the mechanisms by which conflict processing can be influenced by the prospect of reward, with a specific focus on the temporal dynamics of the underlying neural processes.

Using a rewarded version of the color-naming Stroop task, we recently demonstrated that behavioral interference can be diminished by explicitly associating the task-relevant stimulus dimension (font color) with reward in the form of monetary incentives [14]. Specifically, participants were informed that they could win money for fast and correct responses to two specific font colors out of four possible ones that could appear. The results showed that responses to such reward-predictive font colors were not only faster in general, but that the behavioral interference from incongruent word meanings was strongly attenuated in these trials. Results from our corresponding functional magnetic resonance imaging (fMRI) study suggested that these effects arose from modulations in prefrontal regions that have been implicated in cognitive control, as well as in the ventral striatum, which has been commonly associated with the processing of rewards [15]. At the same time, task-irrelevant words that were semantically referring to reward-predictive colors induced greater behavioral-interference effects in this task. These additional behavioral costs were associated with increased activity in the pre-supplementary motor area (pre-SMA), suggesting that salient reward-related word meanings trigger automatic response tendencies that are more difficult to override if they are incongruent with the task goal.

While these findings offer important insights into the neural substrates associated with the effect of reward on conflict processing, they do not provide any information about the temporal characteristics of these neural modulations, which are critical for understanding the underlying mechanisms. In order to better elucidate the temporal and functional dynamics of these neural processes, we performed an analogous version of this rewarded Stroop paradigm while recording event-related brain potentials (ERPs) with the goal of capitalizing on their high temporal resolution for measuring brain activations related to cognitive processes. Specifically, we sought to examine if, and how, reward associations might alter the timing of ERP components associated with different processing stages during this task. On the one hand, we focused on ERP modulations that have been associated with increased attention to reward-related stimuli. Specifically, reward-related cue and target stimuli, as well as reward feedback, have been shown to elicit activity modulations in the 200 to 400 ms time range, including effects on the N200 and P300 components [e.g., [16], [17]–[20]].

Such attentional modulations are likely to occur before or contemporaneously with processes explicitly related to conflict processing, namely the incongruency-related negativity (N_inc_ or N450) and the subsequent late positivity component (LPC). The N_inc_/N450 is commonly observed between 400 and 600 ms when comparing incongruent to congruent trials, has a centro-parietal scalp distribution in the manual-response mode, and is thought to arise in part from medial frontal brain regions [21]–[26]. This component has been related to conflict processing in general, and more specifically to the detection of visual color-word conflict as evoked in the Stroop task, a notion that has received support by recent studies investigating the influence of trial history on conflict processing [e.g., [27]]. The N_inc_/N450 in the Stroop task is typically followed by an LPC, a parietally distributed slow wave that has been associated with more controlled response-selection and conflict-resolution processes [22], [27], [28], as well as with re-current activity modulations in posterior word processing regions [21].

In the context of the present study, we investigated modulations in early attention-related and subsequent conflict-related ERP components to assess the temporal dynamics of reward influences on conflict processing. First, we hypothesized that reward-predictive stimuli would elicit relatively early activity modulations in ERP components implicated in attentional deployment. Increased attention to reward-predictive stimuli might in turn change how conflicting information is processed in the context of reward. Here, several main possibilities are conceivable: (1) conflict-related processes could be unchanged in the context of reward, suggesting that the reward-related behavioral benefits are implemented at a later processing stage; (2) the amplitudes of conflict-related components could be modulated, with their timing being unchanged, which would suggest that reward mainly improves the efficiency of conflict resolution rather than its speed; (3) conflict-related processes might occur earlier in time, suggesting that reward accelerates the access to all stimulus features, task-relevant and task-irrelevant ones; (4) conflict-related processes could be attenuated or abolished, which would suggest that reward leads to a selective prioritization of only the task-relevant inputs early on, rendering conflict processing dispensable. Finally, we wanted to use an equivalent logic to investigate how implicit reward associations to features in the task-irrelevant dimension results in augmented behavioral interference.

## Materials and Methods

### Participants and Paradigm

Fourteen healthy right-handed volunteers participated in the study (mean age ± SD: 22.6±3.5, 10 female). Four additional participants had to be excluded due to artifacts in the EEG recordings (see below). All participants gave written informed consent to participate and were paid a basic amount of $30, plus an average reward bonus of $15. Ethical approval in accordance with the Declaration of Helsinki was granted for this study by the Duke Medical Center Institutional Review Board for human subjects.

**Figure 1:**
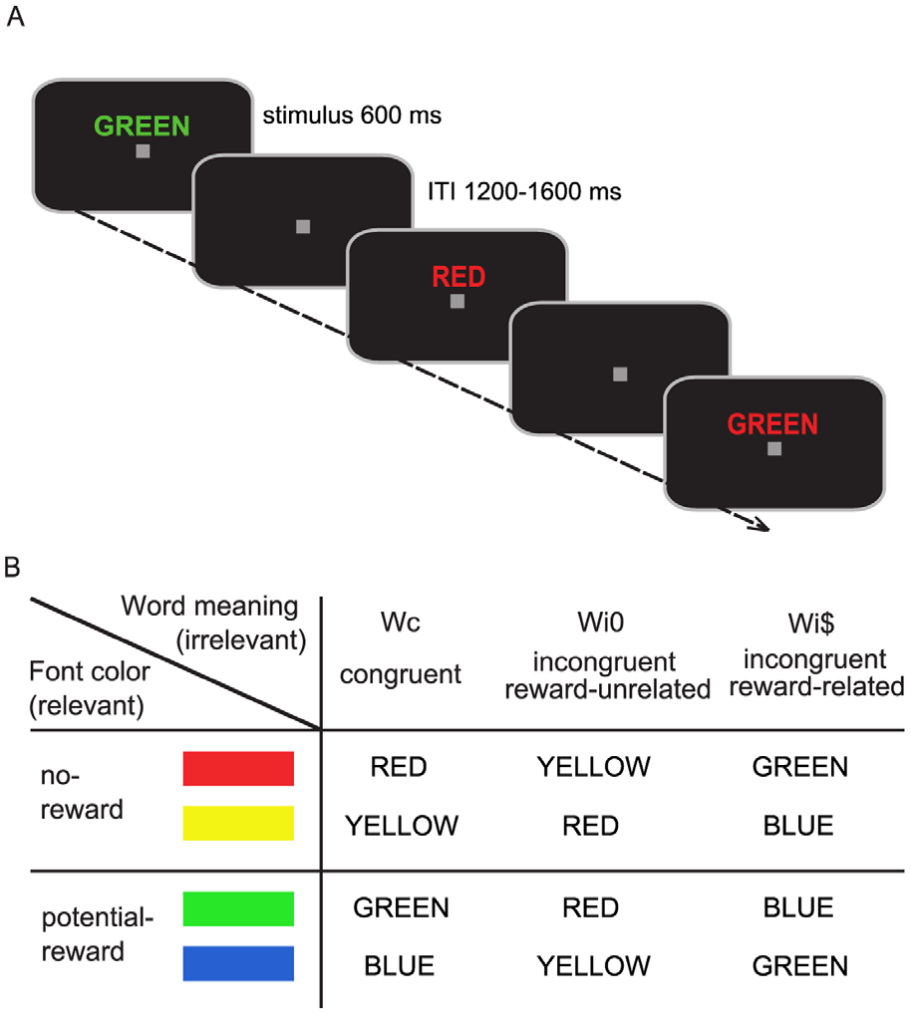
Stimuli and design. (**A**) Colored words were presented for 600 ms on a black background, separated by an inter-trial interval (ITI) of 1200–1600 ms. (**B**) Four font colors and the corresponding word meanings were randomly combined in each trial, thereby creating congruent and incongruent Stroop stimuli. Participants responded to the word’s font color (relevant dimension) while ignoring the word meaning (irrelevant dimension). Responses to a subset of font colors were associated with monetary incentives (e.g., green/blue, *potential-reward trials*), while the remaining colors were not (e.g., red/yellow, *no-reward trials*). Task-irrelevant word meanings could be congruent (*Wc*) or incongruent (*Wi*) to the font color, while the latter could furthermore implicitly refer to potential-reward colors (*Wi$*) or not (*Wi0*).

Participants performed a rewarded color-naming Stroop task (Fig. 1) developed in our earlier behavioral study [14], with some slight adjustments related to the ERP methodology. Throughout the experiment, a small gray fixation square (visual angle 0.3^o^) was maintained in the center of a black screen (Fig. 1A). In each trial a capitalized color word was presented for 600 ms randomly chosen from the following set: “RED”, “YELLOW”, “BLUE”, or “GREEN” (vertical 0.8^o^, horizontal 2.1–4.6^o^). Words were positioned slightly above fixation (0.3^o^) in order not to disrupt word processing by an overlaid fixation point. The words were separated by a variable inter-trial interval (ITI) of 1200 to 1600 ms and were written in one of four font colors (red, yellow, blue, or green). Participants were instructed to respond as quickly as possible on each trial by pressing the button associated with the current font color (task-relevant dimension) while ignoring the semantic meaning of the word (task-irrelevant dimension). Responses were given with the index and middle fingers of the left and right hands, with color-button assignments counterbalanced across subjects. The semantic word meaning *(W)* of a given word could be congruent (*Wc*; e.g., “GREEN” in green font color) or incongruent (*Wi*; e.g., “RED” in green font color) with respect to the font color (Fig. 1B). Note that while additional neutral (or response-ineligible) word meanings were included to match our previous paradigm (e.g., “BROWN” in green font), they were not considered in the analysis. The reason for this decision was that the reward manipulations did not affect the relationship between congruent and neutral trials on the behavioral level [14], indicating that there was no additional reward-related facilitation due to word congruency. Hence, we focused our current analysis entirely on the interference effect and chose the most sensitive and most commonly used contrast with respect to neural activity modulations, namely incongruent minus congruent trials.

Analogous to our earlier behavioral study, responses to two of the four possible font colors were associated with monetary incentives (termed *potential-reward trials;* Fig. 1B), while the remaining two font colors were not (termed *no-reward trials*). Before the experiment, participants were explicitly informed about the specific color-reward associations, e.g., potential reward for words written in blue and green font. These associations remained the same throughout the experiment for each participant, but were counterbalanced across participants. Fast and correct responses in potential-reward trials resulted in a 10-cent gain, while incorrect or slow responses resulted in a 10-cent penalty. In order to keep all participants at a similar reward ratio of 70% throughout the experiment, the response-time (RT) window was adjusted dynamically based on individual performance, leading to a mean monetary gain of ∼$2.50 per run. Specifically, after each trial, the hit rate was updated in the background, and the response time-out for the next trial was shortened or extended by 10 ms if this rate was above or below 70%, respectively. During four 20-second breaks within each run, the updated dollar amount was displayed, serving as intermediate performance feedback. Due to this adaptive routine, participants were only awarded positive amounts as intermediate feedback and were explicitly told that the overall bonus would always be greater than zero. Importantly, all analyses regarding RT and accuracy were based on responses within a window of 200 to 1200 ms after word onset and hence independent of the adaptive ‘time-out’ routine, which was only implemented to generate comparable levels of reward expectation. Responses outside of this window and responses with multiple button presses were considered incorrect (<2% of all trials).

Due to the color-reward associations in the task-relevant dimension, the semantic meaning of incongruent words could implicitly refer to a potential-reward color (labelled *Wi$*) or to a no-reward color (labelled *Wi0*; Fig. 1B). Regardless of the possible implicit relation of the word meaning to the different font-color subsets (potential-reward vs. no-reward), word meanings were always task-irrelevant and not predictive of reward (unless they were congruent with the font-color dimension). Participants were asked to respond to each word as rapidly and accurately as possible.

The averaged RTs and error rates within the potential-reward and no-reward trial types were submitted to 2×2 repeated-measures analyses of variance (rANOVAs) to verify the overall main effects of task-relevant reward and word meaning (*congruent* vs. *incongruent),* averaged across the two incongruent word types *Wi0* and *Wi$*. Additional 2×2 rANOVAs focusing on incongruent trials alone were conducted to investigate the interaction between task-relevant reward (*potential-reward vs. no-reward* trials) and the two incongruent word types (*Wi$* vs. *Wi0*). Paired two-tailed *t*-tests were performed *post hoc* to further analyze the effects underlying any interactions.

### EEG Recordings

Following a short practice run, the actual study was performed. Seated in a sparsely-lit, electrically-shielded chamber, participants completed six experimental six-minute runs. This yielded a total of 480 potential-reward and 480 no-reward trials, with equally distributed word-meaning categories. Simultaneously, EEG was recorded from 64 electrodes mounted in a custom-designed electrocap (Electro-Cap International, Eaton, Ohio), referenced to the right mastoid during recording. Electrode impedances were maintained below 2 kΩ for the mastoids, below 10 kΩ for the electro-oculogram (EOG) electrodes, and below 5 kΩ for all the remaining electrodes. All 64 EEG channels were continuously recorded with a band-pass filter of 0.01–100 Hz at a sampling rate of 500 Hz (SynAmps amplifiers from Neuroscan; El Paso, TX). Blinks and eye movements were recorded by horizontal and vertical EOG electrodes, and participants were additionally monitored online via a video camera in the EEG chamber.

### EEG Analysis

Data were pre-processed using Brain Vision Analyzer (BVA) 1.05 software package (Brainproducts, Munich, Germany). In advance, all trials entailing incorrect behavioral responses were excluded from the analyses. ERP data was partitioned into 1000-ms epochs that included a 200-ms pre-stimulus segment, which was used for baseline correction. Epochs containing eye movements, eye blinks, muscle-related potentials, or drifts were discarded using a semi-automatic artifact-detection routine in BVA (gradient criterion: max. 40 µV between sampling points; difference criterion: max. 120 µV difference within 200 ms). Artifact-free epochs were averaged for each condition of interest and exported to the ERPSS analysis program (University of California, San Diego, CA) for statistical analyses and data plotting.

**Figure 2:**
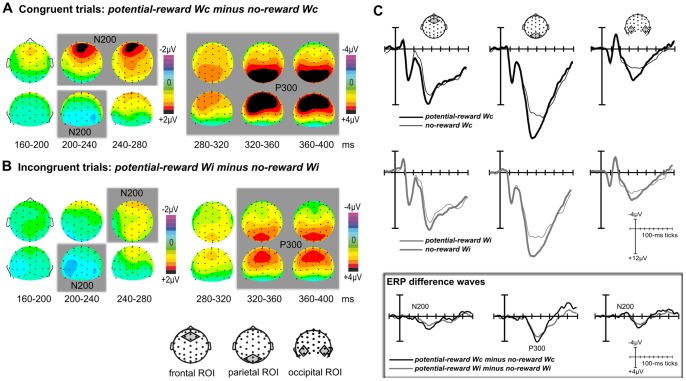
Effects of reward on early stimulus processing. (**A**) Averaged ERP distribution maps of the difference between congruent potential-reward and congruent no-reward trials reveal reward-induced modulations of the frontal N200, the parietal P300, and the occiptal N200 components. (**B**) In incongruent trials (collapsed across the two different word-meaning types *Wi0* and *Wi$*), the reward-induced positive-polarity effects were substantially attenuated. Gray-shaded areas indicate significant mean-amplitude differences in the respective regions of interest (ROIs; p-values<.05). (**C**) ERP waveforms for each condition as well as the respective ERP difference waves are shown for selected ROIs (averaged across channels).

**Figure 3:**
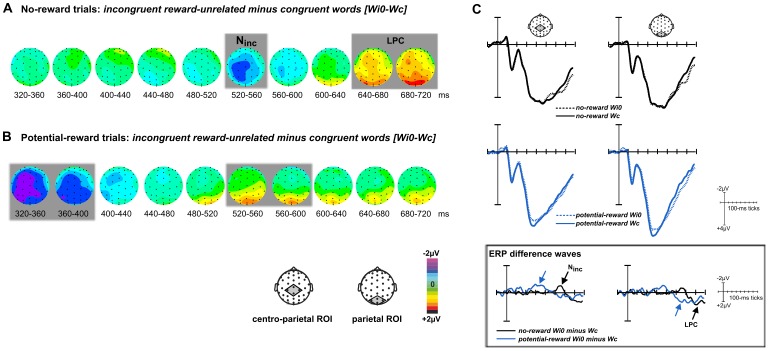
Influences of reward in the relevant dimension on conflict processing. (**A**) Averaged ERP distribution maps of the difference between incongruent and congruent trials (*Wi0-Wc*) in no-reward trials reveal the incongruency-related negativity (N_inc_) over centro-parietal sites followed by the late positivity component (LPC) over parietal sites. (**B**) The analogous comparison in potential-reward trials revealed that the N_inc_ and LPC components were replaced by earlier modulations in the centro-parietal and parietal ROIs. Gray-shaded areas indicate significant mean-amplitude differences in the respective ROIs (p-values<.05). (**C**) ERP waveforms for each condition as well as the respective ERP difference waves are shown for selected ROIs (averaged across channels).

**Figure 4:**
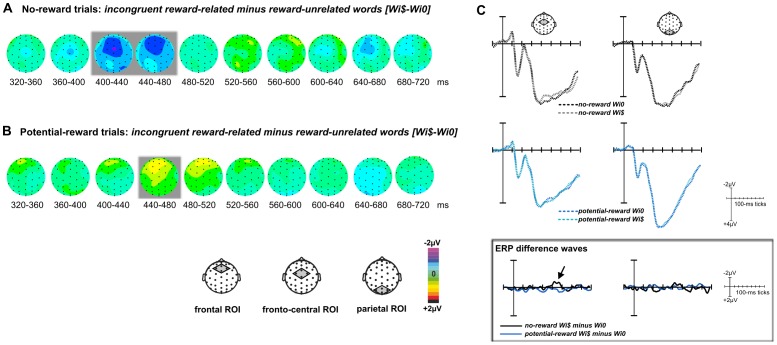
Influences of reward in the irrelevant dimension on conflict processing. (**A**) Within no-reward trials, the direct comparison between two types of incongruent word meanings, i.e., those that were related to reward versus those that were unrelated to reward (*Wi$-Wi0*), revealed a negative component over fronto-central sites. (**B**) In contrast, the analogous comparison in potential-reward trials did not yield any significant differences. Gray-shaded areas indicate significant mean-amplitude differences in the respective ROIs (p-values<.05). (**C**) ERP waveforms for each condition as well as the respective ERP difference waves are shown for selected ROIs (averaged across channels).

In ERPSS, averages of each condition as well as difference waves between conditions were computed across participants. For visualization only, these group averages were low-pass filtered using a non-causal, zero-phase, triangular 17-point running-average filter, which has a half-amplitude cut-off at around 25 Hz. Spherical spline-interpolated topographic voltage maps of the difference waves were created for consecutive 40-ms time windows. Based on the topographic maps, and considering existing ERP studies on reward and conflict processing [e.g., [21], [22], [23]], ERP components within regions of interest (ROIs) consisting of four electrodes each were selected and quantified via paired *t*-tests, comparing the unfiltered mean-amplitude values averaged across 40-ms time windows (frontal ROI: Fz, FCz, F1a, F2a, fronto-central ROI: FCz, Cz, FC1, FC2; centro-parietal ROI: Cz, CPz, CP1, CP2; parietal ROI: Pz, POz, P1a, P2a; occipital ROI: PO7, PO9, PO8, PO10). Note that while some ROIs included overlapping electrodes, statistical comparisons were restricted to non-overlapping ROIs. In order to identify ERP components reflecting the influence of reward in the task-relevant dimension, we compared potential-reward to no-reward trials (Fig. 2). To identify components related to conflict processing, neural responses to incongruent word meanings were compared to those to congruent word meanings (Fig. 3). Finally, to explore the influence of reward information in the task-irrelevant dimension, incongruent reward-related word meanings were compared to incongruent reward-unrelated word meanings (Fig. 4).

## Results

### Behavioral Results

Response times (RTs) and error rates are presented in Table 1. Overall, participants’ responses to the word’s font color were faster in potential-reward trials (mean ± SD: 489±55 ms) as compared to no-reward trials (570±49 ms), reflected statistically in a rANOVA main effect of task-relevant reward (*F*_(1,13)_ = 67.38, *p*<.001). RTs were also significantly modulated by the word meaning, with faster responses on congruent trials compared to incongruent ones, collapsed across potential-reward and no-reward trials (*F*_(1,13)_ = 47.2, p<.001). Furthermore, the two factors of *relevant reward* and *word meaning* exhibited a significant interaction (*F*_(1,13)_ = 6.74, p = .022), reflecting a significantly greater incongruency effect on no-reward trials compared to potential-reward trials (*incongruency effect*, Table 1). In terms of response accuracy, participants committed fewer errors in potential-reward trials as compared to no-reward trials (*F*_(1,13)_ = 12.66, p = .004), and fewer errors in trials with congruent word meanings compared to incongruent ones (*F*_(1,13)_ = 18.39, p = .001). These accuracy effects were also accompanied by an interaction (*F*_(1,13)_ = 10.36, p = .007), again reflecting a greater influence of semantic incongruency in no-reward trials compared to potential-reward trials.

**Table 1:** Performance in the color-naming Stroop task.

		Word meaning (irrelevant)			Incongruency effect	
Font color (relevant)		Wc	Wi0	Wi$	Wi0-Wc	Wi$-Wc
**no-reward trials**	RT ms (SD)	545 (42.7)	575 (51.8)	590 (56.1)	29.3 (19.2)	44.1 (25.7)
	errors % (SD)	9.6 (5.3)	15.9 (8.1)	16.2 (8.5)	6.3 (7.3)	6.5 (5.1)
**potential-reward trials**	RT ms (SD)	474 (50.9)	496 (55.3)	499 (61.7)	21.9 (16.1)	24.7 (21.5)
	errors % (SD)	6.9 (2.5)	7.7 (3.3)	9.6 (6.1)	0.8 (3.6)	2.7 (5.2)

Wc, congruent; Wi0, incongruent reward-unrelated; Wi$, incongruent reward-related.

RT, response time; SD, standard deviation.

To test for differential effects of incongruent word meanings that implicitly referred to a reward-predictive color, we conducted additional 2×2 rANOVAs focusing on incongruent trials alone [(*potential-reward vs. no-reward) x (Wi$ vs. Wi0*)]. We again observed faster responses in potential-reward trials as compared to no-reward ones (*F*_(1,13)_ = 65.67, p<.001), and significantly slower responses to incongruent words that were semantically related to reward-predictive colors (*Wi$>Wi0: F*_(1,13)_ = 5.59, p = .034). An interaction of the two factors was observed at the trend level (*F*_(1,13)_ = 4.4, p = .059). Post-hoc *t*-tests revealed that this effect was driven by significantly greater interference from reward-related words compared to reward-unrelated ones in no-reward trials (*Wi$>Wi0*: *t*_(13)_ = 3.02, p = .01), while no such difference was observed in potential-reward trials (*Wi$>Wi0*: *t*_(13)_ = 0.62, p>.5). Similarly, error rates in incongruent trials were again reduced by reward in the task-relevant dimension (*F*_(1,13)_ = 14.6, p = .002), while incongruent words semantically related to reward had no differential effect on response accuracy (*t*_(13)_<1.1; p>.3), and there was no interaction between the two factors (*t*_(13)_<1; p>.5).

### Effects of Reward Prospect on Early Stimulus Processing

In a first analysis step, we sought to identify early ERP components related to the processing of reward-predictive colors. To this end, we compared congruent potential-reward trials to congruent no-reward trials up until 400 ms after stimulus onset (*potential-reward Wc vs. no-reward Wc*; Fig. 2A). For the comparison of congruent trials, the topographic difference maps show that the prospect of reward induced a frontal activity modulation starting at around 200 ms after stimulus onset, which appeared to result from a smaller, i.e., more positive, frontal N200 component in potential-reward trials (Fig. 2A). This frontal modulation was accompanied by an occipital bilaterally distributed negativity in the same general time range. Subsequently, we observed a large positive deflection over parietal sites in the P300 range for potential-reward trials compared to no-reward ones. The corresponding paired *t*-tests confirmed a significantly greater mean amplitude for potential-reward trials in the frontal ROI in the N200 range (200–240 ms: *t*_(13)_ = 4.60, p<.001; 240–280 ms: *t*_(13)_ = 3.85, p = .002), in the occipital ROI in the N200 range (200–240 ms: *t*_(13)_ = 2.18, p = .048), as well as in the parietal ROI in the P300 range (280–320 ms: *t*_(13)_ = 4.41, p<.001; 320–360 ms: *t*_(13)_ = 5.59, p<.001; 360–400 ms: *t*_(13)_ = 5.61, p<.001).

The analogous comparison was performed for incongruent trial types, collapsed across the two types of reward-related and reward-unrelated incongruent word meanings (*Wi0* and *Wi$*; Fig. 2B). As with the congruent contrast, incongruent trials produced significant mean-amplitude differences in the frontal ROI in the N200 range (240–280 ms: *t*_(13)_ = 4.08, p = .001), in the occipital ROI in the N200 range (200–240 ms: *t*_(13)_ = 2.63, p = .021), as well as in the parietal ROI in the P300 range (320–360 ms: *t*_(13)_ = 2.84, p = .001; 360–400 ms: *t*_(13)_ = 5.61, p<.001). The frontal and parietal effects in this contrast, however, appeared to be substantially smaller as compared to the same contrast for congruent trials (see ERPs in Fig. 2C). In order to further verify the apparent attenuation of the reward-driven components in incongruent trials, we computed paired *t*-tests directly comparing the mean-amplitude values of congruent potential-reward trials and incongruent potential-reward trials for all three components in the significant time windows in Figure 2A. We found that the mean amplitudes of both the frontal N200 component (200–240 ms: *t*_(13)_ = 3.18, p = .007; 240–280 ms: *t*_(13)_ = 2.52, p = .025) and the parietal P300 component (320–360 ms: *t*_(13)_ = 3.37, p = .005; 360–400 ms: *t*_(13)_ = 3.63, p = .021) were significantly reduced in incongruent compared to congruent potential-reward trials. In contrast, the occipital N200 was unaffected by the congruency of the stimulus in these trials (N200: *t*_(13)_<1; p>.5).

### Effects of Relevant Reward on Conflict Processing

The second set of analyses was aimed at illuminating the influence of task-relevant and task-irrelevant reward associations on hallmark ERP components related to conflict processing. To identify such incongruency-related components under the most straightforward conditions, we first compared incongruent reward-unrelated words to congruent ones in no-reward trials (*Wi0-Wc*; Fig. 3A), thereby creating a baseline contrast that was independent of reward in either stimulus dimension. In this contrast, we observed an incongruency-related negative-polarity ERP starting at around 500 ms after stimulus onset, which was followed by a later positivity starting at around 600 ms. The corresponding paired *t*-tests confirmed a significantly enhanced, temporally focal, negative-polarity wave within the centro-parietal ROI between 520 and 560 ms (*t*_(13)_ = 2.20, p = .047), as well as a significant subsequent positive-polarity difference within the parietal ROI between (640–680 ms: *t*_(13)_ = 2.56, p = .024; 680–720 ms: *t*_(13)_ = 3.40, p = .005). These two components likely represent the incongruency-related N_inc_ (or N450) and the subsequent LPC, both of whcih are commonly observed in Stroop paradigms [e.g., [22], [23], [27]].

In contrast, the analogous comparison in potential*-*reward trials (*Wi0-Wc*; Fig. 3B) revealed a large centro-parietal negativity starting at around 300 ms and a parietal positivity starting at around 500 ms. Paired *t*-tests revealed significant differences between incongruent and congruent potential-reward trials in the centro-parietal ROI (320–360 ms: *t*_(13)_ = 3.27, p = .006; 360–400 ms: *t*_(13)_ = 3.18, p = .007), as well as in the parietal ROI (520–560 ms: *t*_(13)_ = 2.42, p = .031; 560–600 ms: *t*_(13)_ = 3.39, p = .005; at trend level 480–520 ms: *t*_(13)_ = 1.89, p = .082). Note that although the centro-parietal negativity in potential-reward trials (Fig. 3B) falls partly in the same time range as the reward-driven P300 that was identified by contrasting potential-reward and no-reward trials (Fig. 2), the distribution is clearly more anterior. Given the distributional similarities between (a) the centro-parietal negativity in incongruent potential-reward trials and the incongruency-related N_inc_ and (b) between the subsequent parietal positivity in incongruent potential-reward trials and the LPC (see ERPs in Fig. 3C), we interpret these modulations as reflecting a temporal shift of conflict-related processes (but see discussion section for possible alternative interpretations).

### Effects of Irrelevant Reward on Conflict Processing

Due to the nature of the Stroop task, colors that were associated with actual reward could also occur in the task-irrelevant dimension of the stimulus (word meaning). Accordingly, we also sought to investigate how these task-irrelevant reward associations would influence conflict-related ERP components, which were assessed by directly contrasting the neural response elicited by incongruent reward-related word meanings relative to incongruent reward-unrelated ones (*Wi$-Wi0*). In no-reward trials, the contrast revealed a negative deflection at around 400 ms (Fig. 4A), which preceded the above-identified N_inc_ component (cf., Fig. 3A) and had a more anterior distribution. The statistical comparison revealed significant mean-amplitude differences within the fronto-central ROI (400–440 ms: *t*_(13)_ = 2.18, p = .048; 440–480 ms: *t*_(13)_ = 2.50, p = .027). No further significant mean-amplitude differences were observed in this comparison (all *t*_(13)_<1; p>.4), except for a trend for a greater positive deflection in the parietal ROI at an earlier time range (200–240 ms: *t*_(13)_ = 2.13, p = .064, not shown in the topographic voltage maps).

In contrast, the analogous comparison between reward-related and reward-unrelated word meanings in potential-reward trials (Fig. 4B) revealed no significant mean-amplitude differences in the centro-parietal and parietal ROIs (all *t*_(13)_<1; p>.6). This lack of modulation in the conflict-related ERP components for potential-reward trials (see ERPs in Fig. 4C) nicely parallels the behavioral pattern, in that no differences in performance were observed between incongruent reward-related and incongruent reward-unrelated words in potential-reward trials. The only significant difference in this comparison was a small positive modulation in the frontal ROI (440–480 ms: *t*_(13)_ = 2.19, p = .047).

## Discussion

Previous work has demonstrated strong influences of the prospect of reward on human performance. These typically beneficial effects appear to rely on attentional-control mechanisms that promote efficient stimulus processing and facilitate the required response [e.g., [5], [15]]. Recent reports, however, have also uncovered detrimental effects of reward associations on performance when they are linked to stimulus inputs that are irrelevant to the task [e.g., [14], [29], [30]].

The present EEG study was aimed at illuminating the temporal dynamics of these opposing reward-related effects in the color-naming Stroop task – i.e., the suppression of incongruency effects by reward associations with the font color (task-relevant dimension) on the one hand, and increased interference resulting from reward associations with the word meaning (task-irrelevant dimension) on the other hand. In a first step, we identified components related to the processing of the stimulus’ reward value by comparing congruent potential-reward trials to congruent no-reward trials (Fig. 2). We found that reward-predictive font colors were associated with differences in relatively early ERP components, i.e., frontal and occipital modulations in the N200 range, and a distributed modulation over parietal sites in the P300 range. The N200-latency modulation over occipital sites may reflect increased attention to task-relevant stimulus features (here, the reward-predictive font color). Similar modulations have been observed in response to emotional stimuli [e.g., early posterior negativity, [31]], as well as to task-relevant features of neutral stimuli [32]. Intriguingly, in this latter study, the occipital negativity associated with the detection of task-relevant stimulus features was accompanied by a frontal positivity, which is highly consistent with the pattern in the present study. The frontal and parietal modulations in the N200 and P300 time range, respectively, are furthermore in line with studies in which participants encountered affective stimulus material [e.g., [31], [33], [34], [35]] and in reward paradigms investigating ERP responses to reward-predicting cues, as well as to rewarded target stimuli [16], [18]–[20]. Moreover, similar modulations in the N200/P300 range are commonly observed in studies focusing on reward feedback [e.g., [17], [36]]. Together, these early activity modulations most likely reflect increased attention to the reward-predictive features in the present study. As the stimuli in potential-reward trials are targets and reward-predictors at the same time, this attentional modulation may be related to both task-related effort to obtain the reward, and the perception of the reward signal itself. When discussing modulations in the P300 component, it is important to consider the potential relationships with response execution [e.g., [37]]. Although there was no correlation between P300 amplitude and RT in the present study, it seems feasible that the larger P300 amplitude in potential-reward trials could at least partly be a reflection of faster responses. On the other hand, the P300 has been shown to be modulated by expected reward in the absence of behavioral responses [e.g., [16], [18]], highlighting attentional modulations of the P300 in the context of reward that are independent of response execution. The question whether the P300 differences between potential-reward and no-reward trials that we observed in the present study arise as a consequence of response facilitation, attentional modulations, or both, cannot be conclusivly answered based on the current data. Interestingly, the reward-related modulations of the frontal N200 and parietal P300, but not of the occipital N200, were diminished in trials in which the word meaning was incongruent to the font color (Fig. 2B vs. 2A). Such amplitude modulations due to stimulus incongruency have been recently reported in the N200/P300 time range [19], and might be directly related to increased attentional demands [38], or to a somewhat decreased reward expectancy in incongruent trials [17] in the present study. Regardless of the exact mechanism, the latency of this incongruency modulation indicates that the semantic meaning of the word is already accessible as early as 200 ms after stimulus onset. Such early access to task-irrelevant features may form part of the mechanism that provides an advantage for conflict resolution, as discussed below.

To explore these beneficial consequences directly, we next focused on the processing of incongruent compared to congruent stimuli (Fig. 3). To this end, we identified hallmark ERP components of conflict processing, namely the centro-parietal N_inc_ and the parietal LPC, by comparing incongruent words to congruent ones in the absence of reward associations in either dimension. While some studies on Stroop interference have observed the N_inc_ to sometimes be more anterior [24], the centro-parietal scalp distribution observed here is consistent with a number of studies using similar stimuli and response settings [21]–[23], [25], [39]. Specifically, the use of a manual-response mode, as employed in the present study, tends to invoke a more posterior N_inc_ as compared to covert or oral responses [for a direct comparison see, [21], [40] by a pre-target cue]. Furthermore, the N_inc_ has been found to be somewhat diminished and to peak somewhat later in experiments with a high probability of incongruent trials [22], [39], an effect that has been attributed to increased levels of proactively sustained attention that reduces the influence of interfering inputs [41], [42]. Hence, the manual-response mode, together with the relatively high number of incongruent trials, likely accounts for the posterior and somewhat smaller N_inc_ in the present study.

Importantly, in the context of reward, the conflict-related components (N_inc_ and LPC) appeared to be virtually “replaced” by very similar, but substantially earlier, activity modulations in the same centro-parietal and parietal ROIs (Fig. 3A vs. 3B). This observation leads us to reject the first two possibilities raised at the end of the introduction, namely (1) a preserved temporal profile and amplitude of conflict-related components, and (2) a preserved temporal profile but modulated amplitude in the context of reward. Rather, consistent with alternative (3) raised in the introduction, reward prospect appeared to modulate the temporal dynamics of conflict-related processes. More specifically, the data suggest that the hallmark components of Stroop interference (N_inc_ and LPC) occur earlier in the context of reward. From a mechanistic perspective, it appears possible that the early attentional facilitation in potential-reward trials leads to an advanced access to all stimulus features, including the accompanying task-irrelevant word meaning, thereby giving conflict processing a head start. Interestingly, substantial reward-triggered latency modulations have been preciously observed in a magnetoencephalography (MEG) study investigating the influence of reward on novelty detection [43]. Assuming that the early centro-parietal negativity is an advanced version of the N_inc_, the amplitude appears to be larger in the context of reward. This amplitude difference could reflect an increased suppression of the interfering input, or alternatively, a modulation of the conflict-detection signal by stimulus saliency – a notion previously suggested in studies investigating conflict in the presence of emotional stimuli [44], [45].

It is important to consider whether these differential inconruency effects could actually reflect a modulation of the P300 component, considering their temporal overlap. It is the case that in the canonical comparison between incongruent and congruent no-reward trials, the extracted conflict-related modulations (N_inc_ and LPC) overlap with the latency range of the P300 wave. However, the N_inc_ and the LPC are widely considered as separate, and separately identifiable, components from the P300 in the Stroop task, and it has moreover been demonstrated that the P300 peak amplitude is typically not significantly modulated by word congruency [e.g., [28], [46], [47]], as it is the case in no-reward trials in the present study (Fig. 3C, top row). Although the putative N_inc_ falls together with the P300 peak in potential-reward trials (Fig. 3C, middle row), the topographic distribution is clearly distinguishable from the classic parietal P300 and shares more similarities with the centro-parietal N_inc._ This notion is not only supported by the preserved topographic distributions of the N_inc_ and the LPC in the presence and absence of reward, but also by the preserved relative delay between these two components. Together, the observed pattern would seem to suggest a temporal shift of the cascade of conflict-related processes, rather than a more classic modulation of the P300. Regardless of the exact neural mechanism, the reward-triggered effects on the ultimate behavioral output are in line with existing cognitive-control models [8], [10], [11]. Specifically, reward may amplify attention to reward-predictive colors and thus lead to a more rapid activation of the associated response pathways. As increased attention to the entire stimulus might provide faster access to the task-irrelevant information as well, including the associated activation of the task-irrelevant response pathway, conflict resolution may occur earlier in these trials.

Although we use the generic term of reward association to describe the current experimental manipulation, it is important to discuss whether the observed behavioral and neural modulations in reward trials are primarily related to the anticipation of reward or possibly to a specific combination of reward and punishment contingencies. First, the bonuses participants could win were an addition to the hourly payment and they were informed that no money would be deducted from this basic payment. More importantly, the adaptive procedure for reward was strongly in the participants’ favor so that all participants not only received a comparable net bonus, but that none of the intermediate feedback screens signaled a loss. This setup makes it rather unlikely that participants actually expected to lose money or that potential-reward trials possibly triggered any other strong negative associations, which would for example be expected for real aversive events such as electrical shocks. Second, the direct comparison between versions of the task that exclusively used reward and those entailing both reward and punishment [14] did not reveal any differences regarding the behavioral benefits. Finally, there is evidence that feedback related to both winning and avoiding-to-lose similarly activate the ventral striatum, a hallmark region for the positive evaluation of rewards [e.g., [48]]. Moreover, in the fMRI version of our paradigm [15], reward trials were associated with neural activity in the ventral striatum, while there was no evidence for activations in regions implicated in the processing of fear and punishment signals, such as the amygdala [cf., [49]]. Based on these considerations, we would argue that the differential effects between potential-reward and no-reward trials arose with little contribution from a fear of punishment, but were rather triggered by the general reward expectation associated with specific font colors.

The present results extend, in important ways, the insights gained in our previous fMRI version of this task [15]. Although those fMRI results identified key regions implicated in cognitive control and reward evaluation, they did not provide insights into the underlying temporal dynamics. Here, by leveraging the high temporal resolution of ERPs, the results are able to strongly implicate accelerated stimulus and conflict processing as the underlying mechanistic change. Note that, in a related fMRI interference task that used cues to signal the availability of reward for a subsequent congruent or incongruent display, it was demonstrated that facilitory effects of reward relied on preparatory influences triggered by the cue, leading in particular to a more efficient suppression of task-irrelevant stimulus information [5]. Considering that in our paradigm the availability of reward was embedded in the target stimulus itself rather than being predicted by a pre-target cue [cf., [5]], preparatory mechanisms are unlikely to account for our results. Rather, it appears that the mechanism of action here is based on within-trial modulations related to the processing of the reward-related stimulus features themselves.

As mentioned above, the beneficial effect of reward-predictive features in the task-relevant dimension stands in contrast to performance detriments induced by reward associations in the task-irrelevant dimension. Specifically, the occurrence of incongruent reward-related information in the semantic dimension of the stimulus resulted in larger incongruency effects and was paralleled by a negative fronto-central ERP component clearly preceding the time range of the more posterior N_inc_. In addition, these stimuli elicited an even earlier marginally significant positive deflection in the N200 range over parietal sites, potentially indexing increased attentional capture. This view is consistent with ERP modulations in the P200 range induced by attentional manipulations during word processing [e.g., [50], [51]], thus underscoring the automatic nature of word-meaning encoding [see, [52]]. These observations support our previous conclusions specifically with respect to pre-SMA activity observed in the fMRI version of this task [15] - activity that we had suggested to be related to an augmented response activation that needs to be overcome [see also, [53], [54], [55]]. The early latency of the fronto-central negativity elicited by task-irrelevant reward-related word meanings (Fig. 4A) supports our previous notion of a highly automatic mapping between salient reward-related words and the associated response. Such reward-triggered response activations would appear to be in line with contemporary conflict models and, more generally, may be a prime example of automatic “response capture” [12]. Importantly, the present data show that while such automatic response activations can be highly efficient when they occur within the task-relevant dimension, they need to be inhibited or overcome if they are not in line with the task goal and can thereby hijack cognitive-processing resources and impairing task performance.

Finally, the current data show an intriguing interaction between the effects of task-relevant and task-irrelevant reward at both the behavioral and neural level. Potential-reward trials were practically immune to the additional behavioral interference from conflicting reward-related word meanings, suggesting that the early reward-induced attentional facilitation may be acting to protect the system from the automatic response capture of reward-related words. Moreover, the temporal cascade of the neural processes appears to hint at a specific cause for this effect. In particular, in no-reward trials, the first clear signature dissociating incongruent reward-related words from incongruent reward-unrelated words was the fronto-central negativity around 400 ms after stimulus onset. In potential-reward trials, however, this effect was not observed, probably because the reward-triggered stimulus-processing cascade was already in full swing by this time, so that even interference from salient word meanings could be successfully overcome.

In conclusion, by measuring EEG in response to potential-reward and no-reward Stroop stimuli we were able to demonstrate that the behavioral facilitation in potential reward trials was associated with early fronto-central and occipital ERP modulations that likely reflect increased attention to the task-relevant stimulus dimension that predicted reward. Moreover, compared to no-reward trials, this attentional enhancement appeared to modulate the temporal dynamics of conflict processing, enabling a reduction of behavioral interference in potential-reward trials. More generally, the data contribute to an understanding of reward-driven behavioral benefits, particularly in situations in which attentional control is essential to overcome conflicting stimulus inputs.
